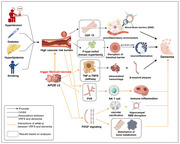# Multiplicative and additive interaction effects of vascular risk factor burden with *APOE* genotypes on dementia: associations and peripheral biological mechanisms based on a large cohort study

**DOI:** 10.1002/alz70861_108162

**Published:** 2025-12-23

**Authors:** Wei Xu, Liangyu Huang, Lan Tan

**Affiliations:** ^1^ Qingdao Municipal Hospital, Qingdao University, Qingdao, Shandong China; ^2^ Qingdao Municipal Hospital, Qingdao, 266071 China; ^3^ Qingdao Municipal hospital, Qingdao university, Qingdao, Shandong China

## Abstract

**Background:**

Gene‐environment interaction is important for understanding precise etiology of dementia. This study aimed to assess the associations of vascular risk factor (VRF) and its interaction by *APOE* genotypes with risk of all‐cause dementia (ACD) and Alzheimer's disease (AD), and to provide cluses of the peripheral biological mechanisms.

**Methods:**

A total of 264,382 participants (median age: 57 years) from the UK Biobank were followed for an average of 13 years. A VRF burden score (VRFS) associated with dementia risk was constructed incorporating hypertension, diabetes, hyperlipidemia, and current smoking. Cox proportional hazards regression with a death‐competing risk model was performed to explore the associations of VRFS with dementia risk, as well as the additive and multiplicative interactions of *APOE* by VRFS. Blood proteomics, bioinformatics, and mediation analyses were applied to investigate the potential mechanisms.

**Results:**

An elevated VRFS was significantly linked to a 33% higher risk of ACD and AD (P < 0.001), and the associations were not confounded by death events (P < 2×10^‐16^). Significant multiplicative and additive interaction effects were detected (P < 0.001). The co‐existence of higher VRFS and *APOE* ε4 produced significantly larger effects on dementia risk than their presence alone (attributable proportion due to Interaction= 0.12). However, the effect magnitudes of VRFS on dementia were enlarged in the presence of *APOE* ε2 (risk elevation ∼70%, *p* < 0.001) than *APOE* ε4 (∼24%). A cluster of biologically connected proteins (82 for ACD and 47 for AD) survived FDR correction and were revealed to mediate the associations of VRFS with ACD (1.5% ∼ 31.5%) or AD (1.3% ∼ 31.3%). These proteins were enriched in pathways such as *p* ‐type trefoil domain superfamily, tumor necrosis factor receptor (TNFR), and negative regulation of bone remodeling for ACD, and TNFR, virus receptor activity, and protease inhibitor for AD. Mediator proteins exclusively in *APOE* ε2 carriers were annotated with disulfide bond, TNFR, as well as the regulation of bone metabolism and hemopoiesis.

**Conclusions:**

*APOE* genotypes significantly interacts with VRFS in the development of dementia and AD.